# Mixed comparison of intervention with eccentric, isometric, and heavy slow resistance for Victorian Institute of Sport Assessment Patella Questionnaire in adults with patellar tendinopathy: A systematic review and network meta-analysis

**DOI:** 10.1016/j.heliyon.2024.e39171

**Published:** 2024-10-29

**Authors:** Yifei Li, Dong Sun, Yufei Fang, Zhenghui Lu, Feicun Shi, Gongju Liu, Yaodong Gu

**Affiliations:** a Ningbo No. 2 Hospital, Ningbo, China; b Faculty of Sports Science, Ningbo University, 315211, Ningbo, China; c Faculty of Engineering, University of Pannonia, 8201, Veszprem, Hungary; d Ningbo Water Sports School, 315211, Ningbo, China; e Zhejiang College of Sports, 311231, Hangzhou, China; f Department of Material Science and Technology, Audi Hungaria Faculty of Automotive Engineering, Széchenyi István University, 9026 Győr, Hungary

**Keywords:** Eccentric, Isometric, Heavy slow resistance, Patellar tendinopathy, Meta-analysis

## Abstract

**Background:**

PT (Patellar Tendinopathy) is a degenerative disorder of the tendons induced via extended overstretching or overuse of the tendons instead than usual inflammation. In the past, humans have centered on a number of strategies of treating PT such as ultrasound and surgical treatment. However, they did no longer genuinely consider the effectiveness of eccentric, isometric, or HSR (Heavy Slow Resistance Training) education for PT; They did now not really outline the stage of PT to beautify the uniformity of the find out about participants; They did no longer immediately examine the affects of isometric, eccentric, and HSR training. This systematic assessment chosen eccentric, isometric, and heavy gradual resistance coaching for the remedy of patellar tendinopathy and their respective prognostic effects will supply valuable, top notch evidence-based insights as properly as vital facts and advice for future scientific administration of patellar tendinopathy.

**Methods:**

A thorough and comprehensive search was conducted across the Web of Science, PubMed, and Scopus databases, encompassing a wide range of relevant journals and sources, in order to perform a rigorous systematic review and network meta-analysis, ensuring the inclusion of all pertinent and high-quality studies. The selected studies satisfied predetermined eligibility requirements, which included: (1) PT patients included in the studies; (2) use of eccentric, isometric, and heavy slow resistance training as interventions; and (3) evaluation of VISA-P (Victorian Institute of Sport Assessment Patella Questionnaire) outcome measures. The effect magnitude was measured using the standard mean difference. The risk of bias inherent in each of the studies that were meticulously selected and included in the comprehensive analysis was rigorously evaluated and assessed using the well-established Cochrane Collaboration Risk of Bias Assessment Tool, ensuring the robustness and reliability of the research findings.

**Results:**

Three scientific databases yielded a total of 1460 studies, of which 7 were included in the final analysis. The findings indicated that eccentric training (0.01 in Rank 1 and 0.06 in Rank 8) is the worst method for increasing VISA-P level in patients with patellar tendinopathy, while moderate resistance slow training (0.25) and Rank 1 and Rank 8 are the best options.

**Conclusions:**

While heavy slow resistance is more suited for attaining long-term improvements in knee function, progressive tendon-loading exercises combined with isometric training or moderate slow resistance training are more beneficial than eccentric training alone. Eccentric training gives a greater range of exercise venues and doesn't require any additional training equipment. The inability to directly compare the effects of heavy slow, eccentric, and isometric resistance training constitutes a significant drawback of this review. This limitation stems from the scarcity of research that compares the outcomes of these various therapeutic approaches. To address this constraint, future research endeavors should strive to conduct comparative studies of these strategies. By doing so, they can aim to bridge this evaluation gap and facilitate a more effective and comprehensive assessment of their respective efficacies.

## 1 Introduction

Instead of conventional inflammation, persistent overstretching or overuse of the tendons causes PT, a degenerative disease of the tendons. It is largely responsible for musculoskeletal diseases, which can affect people of all ages [1]. Patellar tendinopathy, which is usually characterized by localized pain limited to the inferior pole of the patella, is one of the most prevalent causes of discomfort in the anterior knee. Activities involving loads frequently make this pain worse, especially when the knee extensors are stressed. Activities like jumping or squatting that stimulate the patellar tendon to store and release energy can exacerbate the pain considerably [2]. The pain typically worsens with activity and improves with rest. The etiology of PT seems to be multifaceted. There are nine characteristics that may be risk factors for the development of PT. The weight, body mass index (BMI), leg-length difference, foot arch height, quadriceps strength, hamstring flexibility, and vertical jump ability are a few of them. These variables all have the potential to influence the development and severity of physical therapy like eccentric training, emphasizing the intricate relationship between physical attributes and biomechanics [3,4]. As opposed to classic inflammatory disorders, PT is a degenerative ailment primarily brought on by overuse of the knee. The significance of early intervention and customized rehabilitation strategies is emphasized by this. Improved long-term results can be achieved by promoting tendon repair and reducing the progression of PT with early and efficient intervention.

The main goals of treatment for PT are pain relief and tendon restoration, with conservative measures being used initially. Regarding the optimal course of treatment, the medical profession is still split. One frequent conservative treatment that works well is cryotherapy, which mainly reduces pain and suffering due to its analgesic characteristics. Cryotherapy counteracts the neovascularization process, which is thought to be a primary cause of tendinopathy, by applying cold to the affected area. By reducing inflammation and accelerating healing, this treatment strategy hopes to enable a restoration to normal tendon function [5]. The patellar strap helps stabilize the position of the patella, preventing excessive displacement or sliding during movement and reduces excessive friction and pressure on the patella against the femur, thereby helping to alleviate symptoms of PT [6]. Other conservative techniques to support tissue regeneration include extracorporeal shock wave therapy, corticosteroids, nonsteroidal anti-inflammatory medications, and platelet-rich plasma (PRP) [[7], [8], [9]]. Unquestionably, conservative treatment, like eccentric exercise, has the benefit of being very inexpensive and able to be administered by patients at home [10]. But patients with chronic patellar tendinopathy frequently respond less well to eccentric training regimens, and even after they have recovered, they may still feel pain when doing everyday tasks [11]. According to recent study, pain relief and functional performance can be improved with isometric and heavy slow resistance training. Their potential benefits in controlling and healing a variety of musculoskeletal diseases are gaining recognized for these training modalities [[12], [13], [14]]. According to research by Trevor Vander Doelen et al. knee discomfort was quickly reduced by isometric training, and the effects persisted for roughly 45 min after the intervention ended [15]. Different forms of contraction training appear to be a potential approach to transforming the treatment of patellar tendinopathy. Consequently, it makes sense to evaluate their efficacy. The single-leg decline squat was the first exercise in the eccentric training phase. The symptomatic leg was used for the downward component (eccentric phase) and the contralateral leg was mostly used for the ascending component (concentric phase) [16]. The administration of PT now relies heavily on eccentric training. The goal of this therapy is to impart a focused and specific load to the patellar tendon by carefully extending the knee extensor muscles, especially the quadriceps. The benefits of eccentric training in the treatment of PT may stem from this intermittent disruption of blood flow, which lowers pain thresholds; the tendon continuously produces and absorbs forces in a sinusoidal pattern with high-frequency oscillations of tendon forces, which may offer an essential stimulus for tendon reconstruction [17]. To encourage tendon healing, heavy slow resistance training uses eccentric and concentric contractions between 90° knee flexion and full extension. This type of exercise usually consists of deep squats, leg presses, and huck squats. Specialized tools, like a leg press, are needed for this type of exercise. Both tendon hypertrophy and improved tendon mechanical characteristics can be produced by intense resistance exercise [18]. One study found that an eccentric loading regimen is inferior to heavy slow resistance training in terms of long-term response [19]. Typically, 70–80 % of the maximal voluntary contraction is used during isometric training at a knee flexion angle of 60°. It has been shown that isometric training influences changes in the cortex, which relieves pain. Among other things, this includes a drop in intracortical inhibition, which is connected to less discomfort [14].

Research on eccentric training associated with PT has only begun to gain attention since 1989 [20]. Despite this growing interest, clinical practice lacks evidence-based guidelines for managing PT through eccentric, isometric, or HSR training [21]. In addition to failing to specify the stage of physical therapy that would improve participant homogeneity, they failed to assess the efficacy of eccentric, isometric, or HSR training for physical therapy. The effects of isometric, eccentric, and HSR training were also not directly compared by them. Therefore, conducting a thorough literature review on the pros and cons of eccentric, isometric, or HSR training for physical therapy would provide insightful, high-caliber evidence for clinical practice in the future. This review could produce a substantial amount of data and recommendations.

With the goal of directly or indirectly evaluating the various efficacies of eccentric, isometric, and HSR training approaches for the treatment of PT, this systematic review attempts to thoroughly assess and examine these differences.

## 2 Methods

### 2.1 Eligibility criteria

This review gives preference to the Meta-Analyses (PRISMA 2020) guidelines [22] provided by the Cochrane Library and employs the PICOS criteria as the strategy for article inclusion. Participants (P): Adults of both sexes who were athletes with PT were included in the study. Interventions (I): We identified the following effects of eccentric training interventions: Starting eccentric training for physical therapy usually involves the user standing straight on a 25-degree decline board and bearing all of their weight on the leg that is injured. Bending the knee gradually to about 70° is the workout (this step is optional). To help with control and balance, the user uses their other leg to help them return to the beginning position; isometric training: Usually, 70–80 % of the maximal voluntary contraction is used while the knee is flexed to 60°; Training for HSR included three bilateral exercises: the hack squat, leg press, and squat. With all of the participants being patients with patellar tendinopathy, it was vital to keep an eye on the VAS pain score during training and modify the regimen if it went beyond 40. It is therefore common for the training programs of related articles to contain some variances. It is important to note that the use of rehabilitation tools, such as a leg extension machine and a flywheel inertia, was permitted during the interventions. Comparators (C): Our study included studies that contrasted parameters from the Visual Analogue Scale (VAS), Numerical Rating Scale (NRS), and Victorian Institute of Sport Assessment-Patella questionnaire (VISA-P) before and after the intervention. The use of the Bayes theorem is responsible for the viability of indirect comparisons between various interventions. The criteria for the interventions and comparators/controls were in agreement. Outcomes (O): The majority of the time, participant subjective assessments were used to calculate the results. This extensive questionnaire, known as the VISA-P, was created especially to assess the amount of pain and athletic function related to patellar tendon disorders. When a person receives a score of 100 on this quiz, it means they are fully recovered and functioning at their best. The VAS and NRS can be utilized to measure pain even more precisely, offering more information about the intensity of the pain and how it affects daily activities and sports capabilities. The thickness of the patellar tendon can also be determined during the intervention using ultrasound and magnetic resonance imaging (MRI). Study Design (S): Randomized clinical trials were the only kind of studies included in this systematic review.

Exclusion Criteria: (1) Excluded papers included reviews, conference proceedings, case reports, and works written in languages other than English; (2) Excluded studies lacked comprehensive outcome data and statistical analysis. (3) People with diabetes or any other illness that would impair their ability to perform training interventions (such as neurological or cognitive impairment) were not allowed to participate. Nor were people who had recently suffered patellar trauma, such as a fracture, or who had had lower limb surgery or injections within the previous six months. Surgical operations and other intervention methods that don't fit the required standards were not included. (5) Animal-related titles such as “dog” and “mouse” were not included. (6) Other non-training therapies were not included, including knee injections, ultrasonography, and other treatments. The interpretation of abbreviation can be found in Table 1.

**Table 1:** The explanation of abbreviation in this study.

Abbreviation	Definition
CG	Control group
CT	Concentric training
CMJ	Counter movement jump
ET	Eccentric training
ET17	Eccentric training with 17°
HSR	Heavy slow resistance
IFR	Inertial flywheel resistance
MSR	Moderate slow resistance
PTLE	Progressive tendon-loading exercises
PT	Patellar tendinopathy
VAS	Visual analogue scale
VISA-P	Victorian institute of sport assessment patella questionnaire
VISA-L	Victorian institute of sport assessment in left leg
VISA-R	Victorian institute of sport assessment in right leg

Control: Without any intervention.

### 2.2 Information sources

We conducted searches using PubMed, Scopus, and Web of Science databases covering the period from 1957 to 2023 (the latest search: 1 March 2024). This review exclusively considered articles that had undergone peer review and were composed in English during the search, selection, and screening process. Additionally, we thoroughly reviewed the reference lists of selected studies to uncover relevant research in the grey literature. When encountering studies with incomplete data, we reached out to the authors to request the missing information.

### 2.3 Search strategy

Three databases were searched using the following keywords: (“patella∗" OR “knee cap”) AND (“tendinopathy” OR "jumper's knee” OR “tendinosis”) AND (“eccentric” OR “isometric” OR “heavy slow resistance”) NOT (surgery). Following the guidelines, a study search methodology grounded in Boolean logic was employed: (1) The condition-related English keywords “patellar” and “tendinopathy” were chosen from the abstract or title; (2) The terms “dog,” “mouse,” and “mice” were removed from the titles in order to concentrate on human studies; (3) The words “review,” “design,” and “protocol” were removed from the titles in order to focus on potential trials and experimental results; (4) Abstracts that specifically mentioned interventions for trials involving patellar tendinopathy with a control group or indicated that they were randomized clinical trials were taken into consideration; (5) Abstracts that discussed interventions involving heavy, slow, and eccentric resistance exercises for patients with patellar tendinopathy were taken into consideration.

### 2.4 Selection process

Two independent reviewers (Yifei Li and Zhenghui Lu) first assessed all identified trial titles to find suitable trials, then we screened abstracts to maximize the search strategy's sensitivity and specificity. To guarantee the highest level of rigor in the search process, a third independent librarian (Gonju Liu), reviewed and improved the search strategy. This included looking for synonyms and other phrases. Research found in the databases were loaded into EndNote X9 (Thomson Reuters, Carlsbad, CA, USA) for additional processing and duplication elimination. A third author (Yaodong Gu), arbitrated disagreements when a consensus could not be achieved, although Yifei Li and Zhenghui Lu handled the theoretical and content parts of the work.

### 2.5 Data collection process

Two independent investigators (Yifei Li and Zhenghui Lu) carried out the data extraction and analysis, with the provision for a third reviewer (Yaodong Gu) to be consulted in case of any need for consensus.

### 2.6 Data items

The following important variables were covered by the painstakingly retrieved, recorded, and stored data for this systematic review: (1) Participant demographics, including average age, gender distribution, and type of population; (2) Intervention program details, including names, features, and classifications; (3) Outcome measure results, such as VISA-P, VAS, and NRS scores, capturing sample sizes for each paper, data collection timing, and mean outcomes with standard deviations for different endpoints.

### 2.7 Study risk of bias assessment

The assessment of potential bias risk in each included study was conducted using the Cochrane Assessment Tool for Risk of Bias (RoB 1.0) by two authors (Yifei Li and Zhenghui Lu) [23]. According to the recommendations of the instrument, the existence of uncertain or high-risk domains determines the overall bias risk category of a study. A study is deemed to have a “low risk of bias” if it has only one or two ambiguous risk aspects and no high-risk factors. On the other side, a research project is defined as having a “moderate risk of bias” if it has three or more uncertain risk items but no high-risk elements. A study is classified as having “high bias risk” if it contains several high-risk components. Cohen's kappa value was used to gauge the inter-rater agreement between the two independent reviewers. In instances where consensus could not be reached, an impartial arbitrator (Yaodong Gu) was consulted in the event of a disagreement.

### 2.8 Effect measures

Preprocessing of the raw data was required because the included studies may have different outcome measurements. Microsoft Office Excel was used for this (Version 16.0, Microsoft Corporation, Redmond, WA, United States). To enable comparison and analysis between the many research, this software was used to transform all results into a common format, specifically the mean value and its standard deviation (Mean ± SD). Two independent investigators (Yifei Li and Zhenghui Lu) preprocessed and analyzed the data, resolving any discrepancies through discussion. A third reviewer (Yaodong Gu) was available to mediate and ensure consensus when required.

### 2.9 Synthesis methods

We performed a comprehensive review of the database in compliance with the previously defined inclusion and exclusion criteria. The primary results of every study concerning the VAS, NRS, and VISA-P were painstakingly recorded in a neatly arranged table together with all the gathered data. It should be noted that, like many other surveys, the VISA-P was first created with patients who could converse in English in mind. To guarantee the applicability and relevance of the research included in our analysis, this factor was taken into account during the screening procedure [24]. Two studies used the Spanish version of the VISA-P questionnaire and 5 studies used the English version. The reliability and validity of the Spanish and English versions [25] of VISA-P are shown in Table 2. The maximum score of 100 on the eight items of the VISA-P represents a completely asymptomatic and functioning state for the patient. Lower ratings, on the other hand, represent more severe symptoms as well as functional and activity limits. The total of the scores for each question determines the final score. In the supplementary file, you can get comprehensive data for every outcome measure. When details were lacking, we contacted the authors to get them to clarify. In order to guarantee the precision and comprehensiveness of our conclusions, we eliminated from our analysis studies for which data could not be obtained, even upon request. The study was performed using ADDIS (V1.16.8, a sophisticated analytic tool from Drugis.org's Aggregate Data Drug Information System (available at https://addis.drugis.org/#/). We performed a network meta-analysis and computed effect sizes; the findings and accompanying graphics are described in the sections that follow.

**Table 2:** The reliability and validity of the Spanish and English versions of VISA-P.

	Reliability	Validity		
Version	Internal Consistency	Test-Retest	Factor Structure	Percent Variance Explained
English	Pearson r, 0.87	…	…	…
Spanish	Cronbach α, 0.83	ICC = 0.99; 95 % CI: 0.992, 0.996	2-factor solution	76.1 %

ICC: intraclass correlation coefficient.

### 2.10 Reporting bias assessment

Using the Cochrane Collaboration Risk of Bias Assessment Tool, we evaluated the risk of bias in each of the included papers. The results of each study's unique bias risk assessment served as the basis for this analysis. We wanted to improve the validity and reliability of our analysis overall, so we used this tool to find and measure any potential biases that might have affected the results [23].

### 2.11 Network meta-analysis

Network geometry is used in Bayesian simulation models to represent the quantity, kind, and robustness of interventions. The ADDIS data can be used to determine how consistent or inconsistent different strategies are. Every intervention is represented as a node in this network geometry, and the connections among nodes indicate direct comparisons. The number of intervention arms is indicated by the numerical values in each row [26].

The primary goal of the evidence framework consistency assessment, which comprised a thorough evaluation of components like homogeneity, study similarity, and the underlying consistency hypothesis, was to firmly ensure the reliability and resilience of the network meta-analysis. Using the pooled results of several investigations, this procedure made sure that the information was trustworthy and appropriate for use in decision-making [27]. The software's evidence analysis may reveal a closed-loop structure, which could explain discrepancies in the outcomes of combined therapies. Two techniques are used to find such inconsistencies. First, by comparing the standard deviations of random effects between consistent and inconsistent models, the degree of agreement within treatments may be assessed. Determining whether there are notable variations in the outcomes is made easier with the aid of this comparison. Second, node splitting analysis is carried out in order to verify the p-values produced by the software and assess the model's applicability. A significant consensus for the intervention may be indicated by the standard deviations in both models aligning, which adds to our understanding of the constancy of the outcomes. To guarantee the statistical significance of the outcomes, a comprehensive study has been carried out using a complex Bayesian framework and a large amount of computational power. The primary goal of this research was to determine whether the network's split nodes' direct and indirect evidence were consistently consistent with one another. The degree of agreement between the direct and indirect evidence was determined for each node using a Bayesian p-value calculation. The consistency model would be deemed suitable for additional examination if the Bayesian p-value for the data gathered from direct and indirect comparisons was more than the 0.05 cutoff, signifying a lack of significant inconsistency [28]. If there were no contradictions or closed loops in the evidence structure that results from the research, a consistency model can be used to compare the relative efficacy of different treatments. When these discrepancies are absent, the data is deemed trustworthy and coherent, which facilitates precise comparisons across various therapies according to their efficacy as determined by the consistency model [29]. Nevertheless, rank probability plots inside the consistency model may be used to enable network meta-analysis when comparing indirect interventions for various variables in the adjusted research. The relative efficacy of different interventions across multiple factors may be seen using these charts. Researchers can find out which treatments are most likely to be successful based on the facts by utilizing rank probability plots to understand the hierarchy of interventions [30]. To guarantee accuracy in analyzing the ordering and likelihood of interventions, we attempted to verify that the total percentage in each row's columns tallied up to 1.00 (or 100 %). This gave us the capacity to order various interventions according to the likelihood that they would be most or least beneficial. By doing this, it would be easy to determine which treatments had the highest chance of being successful and which had the lowest chance of yielding notable gains.

## 3 Results

### 3.1 Search strategy and information extraction

**Fig. 1:**
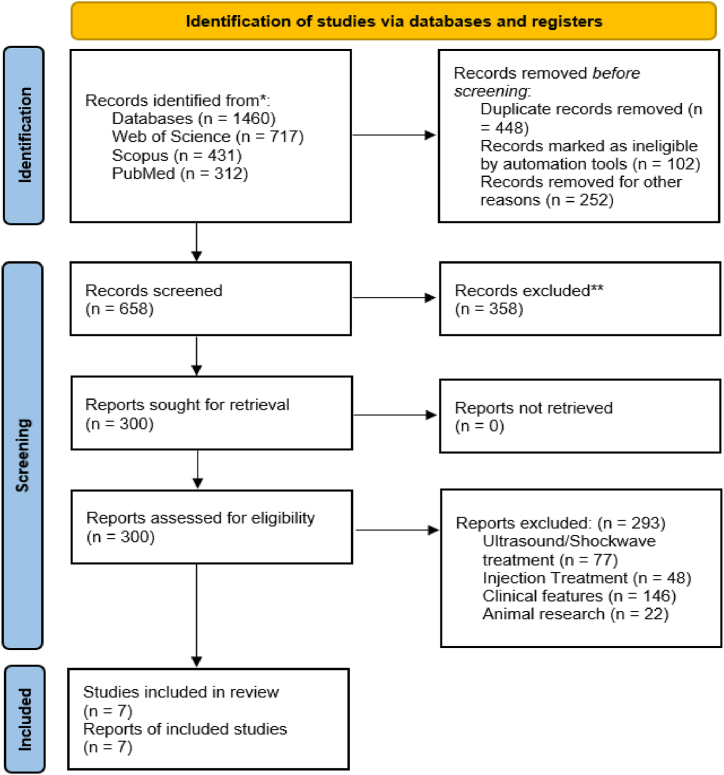
Studies inclusion process.

Three scientific databases were searched thoroughly electronically, yielding 1460 studies. After a thorough screening procedure that evaluated abstracts and titles, a considerable number of articles—1453 in total—were disqualified from consideration for additional review because they did not meet the inclusion criteria or were not relevant. All subjects aged between 18 and 44 years; training interventions included Control Group (CG); Concentric Training (CT); Eccentric Training (ET); Eccentric Training with 17°(ET 17); Heavy Slow Resistance (HSR); Inertial Flywheel Resistance (IFR); Moderate Slow Resistance (MSR); Progressive Tendon-Loading Exercise (PTLE) were included in the final analysis. Fig. 1 provides a detailed illustration of the article screening process, showing how studies are systematically excluded based on predetermined criteria. The study contained just the most pertinent and excellent articles thanks to this strict screening process. Table 3 presents full data on all included research, giving a clear summary of the study features and conclusions.

**Table 3:** The main elements of the included studies.

Studies	Population	Sex	Average Age	Intervention	Outcomes	Main finding
P, Jonsson, 2005	15 athletes with a long duration (mean 17.4 months) of pain from the proximal patellar tendon	13 male 2 female	24.9 ± 8.2	ET	VAS	In eccentric training group, there was a significant decrease in the VAS score at the 12 weeks; VISA score increased significantly; In concentric training group, there was no significant difference in VAS and VISA-P.
				CT	VISA-P	
Kong, 2009	39 recreational male athletes diagnosed with chronic PT	39 male	32.4 ± 8.8	ET	VISA-P	VISA-p improved significantly and similarly in all groups from baseline to 12 weeks.
				HSR	VAS	
Gual 2016	53 players had to be actively competing in an official league, and not currently injured in the lower limb and/or back	27 male 26 female	23.5 ± 4.7	CG	VISA-R	VISA-R and VISA-L displayed no differences across groups at any measurement period.
					VISA-L	
					CMJ	
				IG	Squat-concentric	
					Squat-eccentric	
Ager 2021	42 male athletes, clinical diagnosis of patellar tendinopathy	42 male	30.6 ± 10.0	HSR	VISA-P	The VISA-P score increased in both groups; The NRS score during the SLDS decreased significantly from baseline to 12 weeks for both groups but not from 12 to 52 weeks.
				MSR	NRS	
Breda 2021	76 athletes with clinically diagnosed and ultrasound-confirmed PT	58 male 18 female	24 ± 3.9	ET	VISA-P	The estimated mean VISA-P score improved significantly in both groups.
				PTLE	Return in the desired sports at preinjury level	
Ruff 2021	42 recreational athletes diagnosed with unilateral PT	41 male 1 female	29.6 ± 7.1	HSR	VISA-P	Both groups showed significant improvements in VISA-P and decrease in VAS and PSFS scores from 0 to 12 weeks but no statistically significant between-group difference.
				IFR		
Knez 2023	70 patients diagnosed with PT	49 male 21 female	24.6 ± 6.9	ET 17	VISA-P	Visa-P score improved significantly during the treatment period.
				ET	KOOS score	
					Lysholm/Tegner Activity Scale	
					VAS	

CG: Control Group; CT: Concentric Training; ET: Eccentric Training; ET17: Eccentric Training with 17°; HSR: Heavy Slow Resistance; IFR: Inertial Flywheel Resistance; MSR: Moderate Slow Resistance; PTLE: Progressive Tendon-Loading Exercise; KOOS: Knee Injury and Osteoarthritis Outcome Score questionnaire.

### 3.2 Risk of bias

**Fig. 2:**
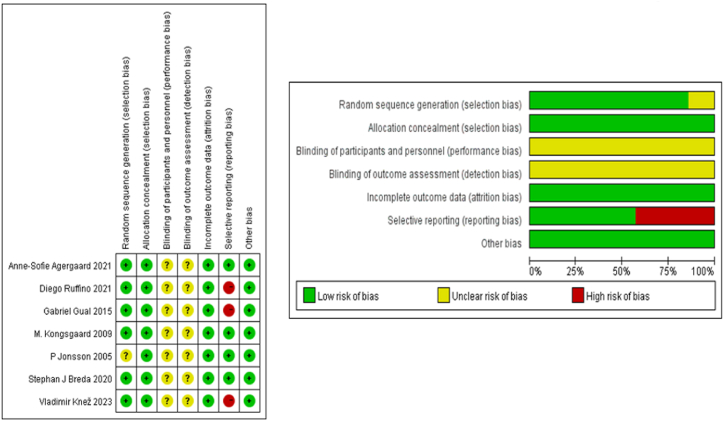
The assessment of bias risk. (a) Summary of bias risk; (b) graph of bias risk.

After a careful evaluation of the seven included studies' likelihood of bias, the researchers came to a conclusion following extensive deliberation. The assessment's overall findings are displayed in Fig. 2. Notably, each study included a sufficient and comprehensive description of the participant concealment and randomization procedures, which enhances the validity and dependability of the results. However, blinding of participants or staff was not adequately described in 50 percent of the studies.

A significant concern was that the studies (7 studies) did not show a rationale for the state of PT in participants. Additionally, due to the nature of the experiment, it was impossible for the participants to remain unaware of the complete experimental process. These aspects are potential areas for improvement in future research.

### 3.3 The network meta-analysis

**Fig. 3:**
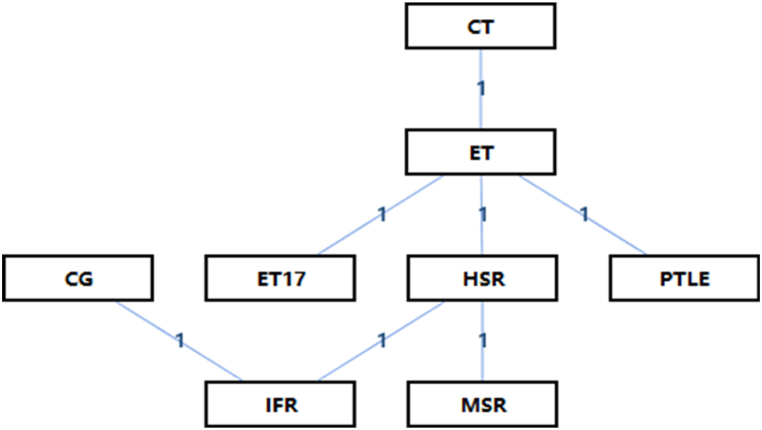
Network structure of loading interventions. These numbers online represent the number of studies that made comparisons of interventions. CG: Control Group; CT: Concentric Training; ET: Eccentric Training; ET17: Eccentric Training with 17°; HSR: Heavy Slow Resistance; IFR: Inertial Flywheel Resistance; MSR: Moderate Slow Resistance; PTLE: Progressive Tendon-Loading Exercise.

The loading intervention's shape as a PT treatment is depicted in Fig. 3. Mixed intervention comparisons are present, as Fig. 3 illustrates. The concept of hybrid intervention comparisons originated from traditional meta-analyses, which involved extending a typical two-arm trial meta-analysis to include a variety of treatment parameters for analysis and comparison. This allowed for the simultaneous synthesis of interventions and outcomes. Indirect comparisons are a part of mixed intervention comparisons.

In mixed comparisons of Control Group (CG); Concentric Training (CT); Eccentric Training (ET); Eccentric Training with 17°(ET 17); Heavy Slow Resistance (HSR); Inertial Flywheel Resistance (IFR); Moderate Slow Resistance (MSR); Progressive Tendon-Loading Exercise (PTLE). For the random effects in the consistency model, the 95 % confidence interval and standard deviation were found to be 1.47 (0.08, 2.92), respectively. This measure sheds light on the diversity of the random effects among various model-included research or data. In the same way, the standard deviation of the random effects and its 95 % confidence interval for the inconsistency model were determined to be 1.55 (0.09, 2.93). Furthermore, the degree of disagreement across studies or observations was indicated by the standard deviation of inconsistency itself, which together with its 95 % confidence interval for the inconsistency model, stood at 1.49 (0.08, 2.93). Moreover, a noteworthy distinction was seen between the random effect standard deviations for the models that were consistent and inconsistent. One probable explanation for the observed variability might be the lack of a clear description of the precise stage of PT employed in the research, which could have undermined the uniformity of the study participants. Furthermore, the approach used limited the capacity to identify the specific cause of the variability by not directly comparing the effects of other training modalities, such as isometric, eccentric, and HSR training.

**Fig. 4:**
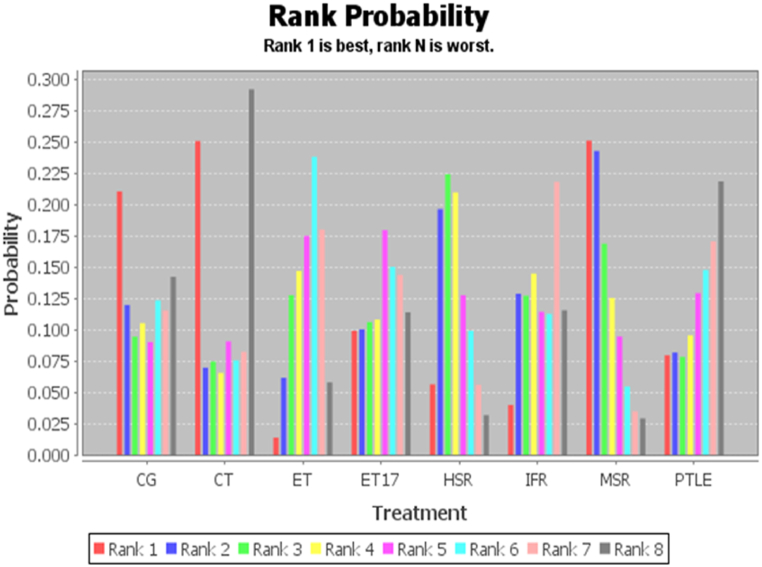
Measurement of VISA-P and ranking of intervention probabilities. CG: Control Group; CT: Concentric Training; ET: Eccentric Training; ET17: Eccentric Training with 17°; HSR: Heavy Slow Resistance; IFR: Inertial Flywheel Resistance; MSR: Moderate Slow Resistance; PTLE: Progressive Tendon-Loading Exercise.

Remarkably, the random-effects standard deviations obtained from the consistency and inconsistency models were discovered to be the same amounts. These parallels imply that the two models' variability is somewhat consistent with one another. This confirmed the viability of the consistency model as a tool for combining data from many research by showing its dependability when used to do network meta-analysis. According to the rankings presented in Table 4, MSR appear to be the preferred intervention when compared with other training methods for VISA-P (0.25 in Rank 1, 0.03 in Rank 8), CT (0.25 in Rank 1, 0.28 in Rank 8), and CG (0.21 in Rank 1, 0.14 in Rank 8). The bar chart presented in Fig. 4 provides a clear visualization of the ranking of various measurements along with their corresponding probabilities. This graphical representation allows for easy comparison and understanding of the relative significance of each measurement. Notably, the better circumstances, the higher VISA-P level. The best rank is one, and the lowest rank is N in the ranked probability plot. The results show that for patients with PT, the methods of elevating VISA-P levels from best to worst are MSR, CT, CG, ET17, HSR, PTLE, IFR, and ET.

**Table 4:** The ranking probability of each comparison between mixed interventions.

Outcome Measures	Interventions	Rank 1	Rank 2	Rank 3	Rank 4	Rank 5	Rank 6	Rank 7	Rank 8
VISA-P	CG	0.21	0.17	0.09	0.11	0.08	0.13	0.11	0.14
	CT	0.25	0.06	0.08	0.06	0.08	0.08	0.07	0.28
	ET	0.01	0.06	0.13	0.15	0.18	0.24	0.18	0.06
	ET17	0.10	0.10	0.11	0.11	0.18	0.15	0.15	0.11
	HSR	0.05	0.20	0.23	0.22	0.13	0.10	0.06	0.03
	IFR	0.04	0.13	0.13	0.15	0.11	0.11	0.21	0.11
	MSR	0.25	0.24	0.18	0.13	0.09	0.05	0.03	0.03
	PTLE	0.08	0.08	0.08	0.09	0.13	0.15	0.18	0.22

CG: Control Group; CT: Concentric Training; ET: Eccentric Training; ET17: Eccentric Training with 17°; HSR: Heavy Slow Resistance; IFR: Inertial Flywheel Resistance; MSR: Moderate Slow Resistance; PTLE: Progressive Tendon-Loading Exercise.

## 4 Discussion

In order to conduct an indirect comparison of diverse training therapies utilized in physical therapy, this review adopted the network meta-analysis method. The aim of the study was to comprehensively evaluate the impacts of various training modalities and intensities on patients undergoing physical therapy. Across 7 randomized controlled trials, 7 distinct interventions were categorized and analyzed: MSR, CT, CG, ET17, HSR, PTLE, IFR, ET. The participants were all included individuals with PT.

Mechanical factors linked to PT encompass either degenerative alterations caused by stress shielding or tendon degradation due to compressive strains [31]. It is usually detected by histology [32,33] and soft tissue findings [34,35]. Many individuals suffer from PT, which is thought to stem from the increased eccentric force exerted on the tendon. This force can be up to three times greater than the concentric force in certain situations, leading to microtears [36]. It is hypothesized that tendon overload may arise from a blend of external factors like floor surface and internal factors such as misalignment [37,38]. The interplay of these factors ultimately dictates the likelihood of a player developing patellar tendinopathy.

In this review, we found that VISA-P was reported in 7 studies. In 7 of the studies, there was a notable increase in VISA-P compared to baseline following training (p < 0.05). The findings indicate that training is beneficial for patients with stable chronic conditions. This conclusion is substantiated by the generally positive impact of training on PT across the included studies. Moreover, there were minimal reports of adverse effects associated with training. Following varied intensity and duration of training, VISA-P showed significant improvements in various patients in these studies. The most effective training intervention was MSR. It is important to note that the Blank, represented by CG, does not consistently rank the lowest in terms of increasing VISA-P levels. According to our findings, ET appears to be the least effective in increasing VISA-P levels. In one study where inertial flywheel was employed [12], the VISA-P score of the participants did not show significant changes following the intervention period, possibly due to its low intensity. Additionally, even in the blank control group, there were notable gains in the VISA-P score. According to the results of our investigation, the inertial flywheel resistance intervention effect was not as successful as it was in the blank control group. This can be because there hasn't been enough study done on other intervention strategies (such seasonal training or the ability of each participant to heal on their own in the blank control group). Further studies are required to examine the impact of each participant's individualized training during the intervention, the various phases of physical therapy, and the recovery levels of each individual on the results.

It is showed that most evidence suggests that training intervention has a positive impact on VISA-P scores in patients with PT. Nevertheless, excessive training may lead to short-term patellar tendon pain, thereby affecting daily life and the recovery process. Throughout all the studies, participants experienced only mild discomfort, indicated by a maximum VAS score of 30. This means that most training interventions for PT allow for the occurrence of pain during training, but the pain is typically within a tolerable range [10]. As a result, depending on the situation and the rehabilitation plan, it is vital to provide training for PT patients that is at the proper intensity. Inquiring about the patient's level of pain during training is also crucial; if the pain gets intolerable, the training regimen should be modified. We will delve deeper into the research and talk about the results in the following parts.

### 4.1 Eccentric

Unquestionably, eccentric training has the benefit of being a relatively affordable therapy option that patients can administer themselves at home. There are 4 studies that employed eccentric training as one of the intervention methods, all using a slanted board with an inclination angle of 25°. Eccentric training is prescribed differently, but most studies use three sets of 15 repetitions twice a day to treat PT during intervention varing from 12 to 24 weeks [19,[39], [40], [41]]. While standing on a descent board, painful eccentric quadriceps training is superior to painful concentric quadriceps training in terms of reducing tendon pain and enhancing function during activity, according to an article analyzing eccentric and concentric training for PT pain and improving knee function [40]. Four of the 7 studies assessed patellar tendon function through various outcome measures, such as VISA-P scores, VAS, KOOS score, Lysholm/Tegner Activity Scale. All eccentric training groups improved their VISA-P scores from baseline to a similar level or higher compared to other active intervention types. However, Stephan J Breda et al. [39] demonstrated that the estimated mean VISA-P score improved significantly from 56 (95 % CI 52 to 61) at baseline to 84 (95 % CI 79 to 89, p < 0.001) at 24 weeks in the PTLE group (which includes isometric, isotonic, explosive training, and project-specific training); from 57 (95 % CI 53 to 62) to 75 (95 % CI 69 to 82, p < 0.001) in the EET group; Although eccentric training has being widely recognized as a beneficial recovery method, eccentric training is less effective than other training interventions that incorporate multiple training methods [19]. Based on our current research findings, the majority of incline board angles are set at 25°. However, one study compared the effects of 17° and 25° incline boards and found no significant difference in VISA-P scores between groups (17°: 53.54 ± 12.29, 81.74 ± 24.59; 25°: 52.77 ± 12.33, 78.74 ± 22.73. 0 week–12 week, mean ± SD) [41]. It can be seen that the decline board angle does not have a significant impact on the effectiveness of eccentric training. Although eccentric training may provide favorable outcomes, the lack of long-term studies (>24 weeks) precludes strong conclusions about its long-term effectiveness. Further research should focus on eccentric training as a prolonged intervention to investigate its long-term effectiveness; It can be compared to a wider range of training interventions to fully assess the effects of eccentric training.

### 4.2 HSR

To promote tendon healing, hip-flexor flexion and extension exercises, or HSR training, alternate between eccentric and concentric contractions. According to a study by Kongsgaard, M. et al., muscle fibril morphology alterations would indicate an increase in muscle fibril quantity, which would improve the rehabilitation outcomes of PT patients. These changes also emphasized the good clinical effects of HSR training [13]. All three studies showed significant improvements in function and reduction in pain after 12 weeks of follow-up, especially after 52 weeks. Nevertheless, one report showed a prominent increase in VISA-P scores in the HSR and MSR groups after intervention, but the HSR group's scores were not as good as those of the MSR group [42]. HSR typically consists of deep squats, leg presses, and huck squats, with four sets of each training, repeated 6–15 times per week. In order to obtain a high resistance, HSR requires the use of specialized equipment such as leg press machine. HSR were performed two to three times per week with the intervention between 6 and 12 weeks. All three studies showed statistically significant improvements in VISA-P scores at 12 and 52 weeks after intervention (p < 0.05). For example, Diego Ruffino et al. [43] recruited 42 amateur athletes with unilateral PT and randomized them into either the inertial flywheel resistance or the HSR group. After the intervention, the VISA-P scores were as follows: HSR group (52.3 ± 13.3 at 0 weeks, 64 ± 12 at 6 weeks, 75.1 ± 11.4 at 12 weeks) and IFR group (49.7 ± 16.8 at 0 weeks, 63.3 ± 13.3 at 6 weeks, 71.9 ± 18.5 at 12 weeks). At the same time, Romero-Rodriguez and colleagues documented a 60 % reduction in pain (p < 0.01) following a 6-week intervention, with outcomes continuing for a 12-week follow-up [44]. We can see that HSR has a superior effect in promoting recovery and long-term pain reduction from PT. However, the convenience of HSR is not as good as that of eccentric training due to the need for special equipment to assist with the training. There is a lack of intervention outcome studies on HSR training in patients at different stages of PT. In the future, HSR training can be performed on patients with different stages of PT to obtain more objective and comprehensive experimental conclusions.

### 4.3 Isometric

Individual isometric training, which was typically paired with other training modalities such isotonic, explosive strength training, and specialized training (PTLE), was not accessible in the included research. Isometric training, a form of resistance exercise, is commonly conducted using a Biodex or leg extension machine. It involves holding a muscle contraction at a specific joint angle, such as 60° knee flexion, at 70–80 % of maximal voluntary contraction for 5 sets of 45 s. A trial incorporating isometric training showed notable improvements in VISA-P scores from baseline, highlighting its effectiveness in enhancing knee function and reducing pain. Training regimens that are isometric and isotonic can help athletes with PT (less than four weeks) feel less pain and perform better [45]. Presently, there exists a significant study vacuum on the relative efficacy of isometric training in comparison to alternative training methods for mitigating patellar tendon discomfort and improving functional levels in athletes, both in and out of season. Determining the best training regimen for patellar tendon problems and maximizing sports performance is difficult due to a paucity of studies. Further research should focus on in-season and out-of-season differences in athletes to comprehensively analyze the benefits of isometric training.

It is not obvious whether intervention may be better at managing PT because this review is limited by its inability to directly compare the effects of isometric, eccentric, and HSR training. This restriction results from the paucity of research that compares the outcomes of these therapies. Future investigations should try to compare these strategies in order to close this gap and enable more accurate evaluation. Moreover, most of the studies lacked a rationale for sample size computations, which may have implications for clinical relevance.

## 5 Conclusion

This study analyzed the findings of different training interventions for the management of patients with PT. The results of these studies were consistent with previous relevant studies. The results suggest that eccentric, HSR, and isometric training are useful in reducing patellar tendon pain. The PTLE that includes isometric training or moderate slow resistance is more effective than eccentric training alone. The HSR is better suited to achieve long-term improvements in knee function. Eccentric training has unrivaled convenience compared to isometric and HSR training, as it does not require additional training equipment and offers a more varied choice of locations. Patient-specific interventions need to be selected. The development of efficient training regimens suited to different phases of PT problems need to be the top priority for future research projects. Distinguishing between in-season and out-of-season variations in athletes is critical, as is evaluating the long-term effects of isometric, eccentric, and HSR training regimens. This will make it easier to determine which training method gives PT patients the best clinical results.

### 5.1 Other information

This systematic review adhered strictly to the guidelines outlined in the PRISMA 2020 statement, ensuring comprehensive and transparent reporting of the method, result, and conclusion in accordance with the latest standards in systematic review methodology [22]. Two independent writers (Yifei Li and Zhenghui Lu), collaborated closely to develop and endorse the screening criteria for the literature, ensuring that the established guidelines were meticulously crafted. They also jointly formulated the search methodologies for the project. The review was not registered and the data was not pre-processed before importation.

## CRediT authorship contribution statement

**Yifei Li:** Writing – review & editing, Writing – original draft, Methodology, Formal analysis, Conceptualization. **Dong Sun:** Writing – review & editing, Conceptualization. **Yufei Fang:** Resources. **Zhenghui Lu:** Writing – review & editing, Writing – original draft, Methodology. **Feicun Shi:** Investigation. **Gongju Liu:** Data curation. **Yaodong Gu:** Supervision, Funding acquisition.

## Availability of data, code and other materials

All data from included studies that used in this review can be found in Web of Science, Scopus, and Pubmed database as well as supplementary file.

## Funding

This study was sponsored by Zhejiang Province Key Research and Development program of “Pioneer” and “Leader” (2023C03197), Zhejiang Province Science Fund for Distinguished Young Scholars (Grant number: LR22A020002), Ningbo Key Research and Development Program (Grant number: 2022Z196), Zhejiang Rehabilitation Medical Association Scientific Research Special Fund (ZKKY2023001), Research Academy of Medicine Combining Sports, Ningbo (No.2023001), the Project of Ningbo Leading Medical &Health Discipline (No.2022-F15, No.2022-F22), 10.13039/100007834Ningbo Natural Science Foundation (Grant number: 2022J065, 2022J120) and K. C. Wong Magna Fund in 10.13039/501100004387Ningbo University.

## Declaration of competing interest

The authors declare that they have no competing interests.

## Abbreviations:

CG: Control Group;
CT: Concentric Training
ET: Eccentric Training
ET17: Eccentric Training with 17°
HSR: Heavy Slow Resistance
IFR: Inertial Flywheel Resistance
MSR: Moderate Slow Resistance
PTLE: Progressive Tendon-Loading Exercise
KOOS: Knee Injury and Osteoarthritis Outcome Score questionnaire.
